# Disulfidptosis-associated long non-coding RNA signature predicts the prognosis, tumor microenvironment, and immunotherapy and chemotherapy options in colon adenocarcinoma

**DOI:** 10.1186/s12935-023-03065-8

**Published:** 2023-09-27

**Authors:** Weijie Xue, Kang Qiu, Bingzi Dong, Dong Guo, Junhua Fu, Chengzhan Zhu, Zhaojian Niu

**Affiliations:** 1 Kumamoto University Graduate School of Medical Sciences, Kumamoto, Japan; 2 Department of Gastrointestinal Surgery, The Affiliated Hospital of Qingdao University, No.16 Jiangsu Road, Qingdao, 266003 China; 3 Department of Hepatobiliary and Pancreatic Surgery, The Affiliated Hospital of Qingdao University, No. 16 Jiangsu Road, Qingdao, 266003 China; 4 Department of Endocrinology and Metabolism, The Affiliated Hospital of Qingdao University, 16 Jiangsu Road, Qingdao, 266003 China; 5 Department of Operation Room, The Affiliated Hospital of Qingdao University, No.16 Jiangsu Road, Qingdao, 266003 China

**Keywords:** Colon adenocarcinoma, Disulfidptosis, lncRNA, Prognostic, Tumor microenvironment

## Abstract

**Background:**

Disulfidptosis is independent of apoptosis, ferroptosis, and cuproptosis and is associated with cancer progression, treatment response, and prognosis. However, the predictive potential of disulfidptosis-associated lncRNAs in colon adenocarcinoma (COAD) and their features in the tumor immune microenvironment (TIME) require further elucidation.

**Methods:**

RNA transcriptome, clinical information, and mutation data of COAD samples were obtained from the TCGA database. The risk model was first constructed by co-expression analysis of disulfidptosis genes and lncRNAs, and prognostic lncRNAs were screened using Cox regression, followed by least absolute shrinkage and selection operator analysis. Enrichment analyses were performed to explore the underlying biological functions and signaling of model-associated differentially expressed genes (MADEGs). Moreover, TIME of MADEGs was analyzed to assess the immunotherapy. Finally, the expression levels of the lncRNAs were verified by taking specimens of patients with COAD from the Affiliated Hospital of Qingdao University.

**Results:**

We constructed a prognosis-related risk model based on four disulfidptosis-associated lncRNAs (ZEB1-AS1, SNHG16, SATB2-AS1, and ALMS1-IT1). By analyzing the survival of patients in the whole, training, and test groups, we found that patients with COAD in the low-risk group had better overall survival than those in the high-risk group. Validation of the model via Cox analysis and clinical indicators demonstrated that the model had a decent potential for predicting the prognosis of patients with COAD. Enrichment analyses revealed that the MADEGs were related to disulfidptosis-associated biological functions and cancer pathways. Furthermore, patients with COAD in the high-risk group had more positive responses to immune checkpoint inhibitors (ICIs) than those in the low-risk group, as confirmed by TIME analysis. ZEB1-AS1, SNHG16, and ALMS1-IT1 were expressed at higher levels in tumor samples than those in the corresponding paracancerous samples (*p* < 0.05), whereas SATB2-AS1 was upregulated in the paracancerous samples (*p* < 0.05).

**Conclusions:**

This signature may guide prognosis, molecular mechanisms, and treatment strategies, including ICIs and chemotherapy, in patients with COAD.

**Supplementary Information:**

The online version contains supplementary material available at 10.1186/s12935-023-03065-8.

## Introduction

Colon adenocarcinoma (COAD) has the third highest incidence rate worldwide, which is second only to lung cancer in terms of cancer mortality [1]. Although the mortality rate of patients with COAD has decreased with improvements in treatment technology in recent years, COAD remains a serious threat to human health worldwide owing to its metastasis, drug resistance, and tumor recurrence features [2–4]. Despite the similarities between COAD and other tumors, the underlying pathogenesis and molecular mechanisms are not sufficiently understood. Therefore, an in-depth exploration of the mechanisms of COAD occurrence and development is urgently required to improve the efficacy of COAD and develop individualized protocols regarding prognostic assessment and treatment options for patients with COAD.

Disulfidptosis, which is independent of the currently existing programmed cell death process, is a rapid mode of cell death caused by disulfide stress resulting from excessive intracellular cystine accumulation [5], during which cell death typically occurs under glucose starvation conditions [5]. It has been revealed that in glucose-deficient SLC7A11-high cancer cells, a large accumulation of disulfide molecules leads to abnormal disulfide bonding between actin cytoskeletal proteins, disrupting their organization and eventually leading to actin network collapse and cell death [5]. Many cancer treatments kill cancer cells through apoptosis [6–8]. However, several cancer cells have developed mechanisms to evade apoptosis, leading to treatment resistance and disease recurrence [9]. These findings suggest that targeting disulfides warrants further investigation as a potential cancer treatment option.

LncRNAs are a category of non-coding RNAs that are over 200 nucleotides in length. Their molecular functions include, but are not limited to, the regulation of various biological processes, such as transcriptional stability, translation, and cell signaling [10–13]. LncRNAs can be transcribed but not translated into proteins, perform their various biological functions only at the RNA level, and are closely related to various human diseases. Numerous lncRNAs have been reported to be aberrantly expressed in COAD and function as oncogenes by affecting the biological functions of COAD cells, such as proliferation, metastasis, and epithelial–mesenchymal transition, through various mechanisms [14–16]. Additionally, lncRNAs have been associated with tumor immunotherapy and drug resistance [17–19].

The effects of disulfidptosis-associated lncRNAs on COAD prognosis, tumor immune microenvironment (TIME), and chemotherapeutic agents remain unclear. Therefore, our study sought to explore the contribution of disulfidptosis-associated lncRNAs in COAD based on bioinformatics analysis and to develop a model to assess patients’ prognosis, TIME, and sensitivity to immunization therapy and chemotherapeutic drugs.

## Methods

### Data collection and collation

The RNA transcriptome, clinicopathological, and gene mutation annotation information of the COAD samples were retrieved and downloaded from the TCGA database (https://gdc.cancer.gov/). Ten known disulfidptosis-related genes were validated by Liu et al. [5].

### LncRNA screening and establishment of the model

A total of 381 patients were first discretionarily divided into training and test sets using the caret package in R. Next, a Spearman correlation analysis was performed using the ggplot2, ggalluvial, and dplyr R packages (*p* < 0.001, |correlation coefficient ≥ 0.4|) to screen for disulfidptosis-associated lncRNAs and visualize these in a Sankey diagram. Univariate Cox (UniCox) regression analysis in the training group was used to screen for disulfidptosis-associated lncRNAs related to overall survival (OS), the results of which were displayed using a forest plot. The lncRNAs obtained from the above steps were further analyzed using a least absolute shrinkage and selection operator (LASSO) analysis to verify the optimal OS-associated lncRNAs and create a prognostic signature. The LASSO logistic regression model was constructed using the glmnet package in R. The model was calculated as follows: ${\text{risk score}}\; = \;\sum {i\; = \;{\text{LnCoef}}\;(i)\; \times \;{\text{EXP}}(i)}$. Patients were divided into low- and high-risk groups based on the median risk score.

### Validation of the model

Kaplan–Meier, receiver operating characteristic (ROC), and consistency index (C-index) curves; forest plots for univariate analysis; and a nomogram were produced based on survival, caret, pheatmap, timeROC, survminer, regplot, pec, and dplyr R packages to validate the credibility of the model for predicting clinically relevant indicators in the whole group patients with COAD. Classification of the expression patterns of disulfidptosis-associated lncRNAs in COAD samples was performed using principal component analysis (PCA), and the spatial dispersion of samples from the two risk groups was visualized using the limma and scatteredplot3 R packages.

### Function and pathway analyses

The differentially expressed genes (DEGs) of patients in the two risk sets were obtained using the R package limma, and gene ontology (GO) functional enrichment and Kyoto Encyclopedia of Genes and Genomes (KEGG) pathway enrichment analyses of these DEGs were performed using the clusterProfiler, org.HS.eg.db, and enrichplot packages. The DOSE package in R was used to perform gene set enrichment analysis (GSEA) to distinguish between the functions and pathways of the two risk sets, cutoff values were taken as the absolute value of logFC > 1, FDR < 0.25, and p-value < 0.05.

### Analysis of the immune landscapes in the two risk sets

The tumor microenvironment was analyzed using the estimate package in R, according to the ESTIMATE algorithm [20] and the variances between the two risk sets were analyzed via the reshape2 and ggpubr R packages (estimate score = stromal score + immune score). To determine the immunological profiles of the 381 samples, expression information was imported into CIBERSORT using the limma, e1071, parallel, and preprocessCore packages in R to assess the percentage of immunocyte infiltration. To explore the composition of immunocytes in the different risk groups, the allocation of immunocytes in the different risk groups was compared using the Wilcoxon test via the limma, reshape2, and ggpubr R packages. In addition, single-sample GSEA (ssGSEA) was performed using the GSVA, GSEABase, ggpubr, and reshape2 R packages to analyze the differences in immune-related functions between the two risk sets.

### Mutations of DEGs and analysis of tumor mutation burden (TMB) and immune checkpoints

Perl was used to extract and process somatic mutation files. The Maftools package in R was used to draw a waterfall plot of the DEG mutations in patients with COAD from the two different risk sets. Differences in TMB and survival between the two risk sets were analyzed using R. In addition, the expression levels of three immune checkpoints (PD1, PD-L1, and CTLA4) were compared between the two risk sets.

### Chemotherapy agent prediction

The half-maximal drug inhibitory concentration (IC50) values of the chemotherapeutic agents were assessed using the limma, oncoPredict, and parallel R packages.

### Experimental validation of the lncRNAs in the model

Tumors and paired paracancerous tissue samples were collected from 15 patients who underwent colon cancer resection at the Affiliated Hospital of Qingdao University. All patients were pathologically confirmed to have COAD and did not receive any preoperative tumor-related treatments. All samples for this study were obtained with informed consent from each patient, authorized by the ethics committee of the hospital, and conducted in accordance with the Declaration of Helsinki. Quantitative real-time PCR (qRT-PCR) was used to evaluate lncRNA expression.

Total RNA was extracted from the collected tissue samples using RNA-easy Isolation Reagent (Vazyme, China) according to the manufacturer’s protocol and then reverse transcribed into complementary DNA (cDNA) using HiScript III RT SuperMix for qPCR + gDNA wipe (Vazyme, China). A mixture of ddH_2_O, primers, cDNA, and ChamQ Universal SYBR qPCR MasterMix (Vazyme, China) was prepared according to the manual, and qRT-PCR was performed using a PCR reaction detection system with the following procedures and parameters: pre-denaturation (95 °C for 120 s), denaturation (95 °C for 20 s), annealing (60 °C for 20 s), and extension (72 °C for 30 s). A total of 40 cycles were performed. The data were normalized using a control group of β-actin. The primer sequences are shown in Table 1.

**Table1:** lncRNA PCR primer

Name	Primer sequence
ZEB1-AS1	F: CGAATCCCTTCCTCCTCTCC
	R: TCGTCTTAGCCCTTTCCGTT
SNHG16	F: AGCAGAATGCCATGGTTTCC
	R: GGTCAATTTAGGGCACGGTCT
ALMS1-IT1	F: GCAGTGGTTCTTGACGGGTA
	R: CAGTCCAGCCTGGGCAATAA
SATB2-AS1	F: CGAATCCCTTCCTCCTCTCC
	R: TCGTCTTAGCCCTTTCCGTT
β-actin	F: CCTCTCCCAAGTCCACACAG
	R: GGGCACGAAGGCTCATCATT

## Statistical analysis

The two groups of continuous variables were compared using *t*-tests. The chi-square test was performed for the classified variables. Cox regression analysis was used for the univariate and multivariate survival analyses. The log-rank test was used to analyze the OS data. All data were analyzed using R 4.2.2 or GraphPad Prism 7.

## Results

### Disulfidptosis-associated lncRNAs in patients with COAD and establishment of the risk model

**Fig. 1:**
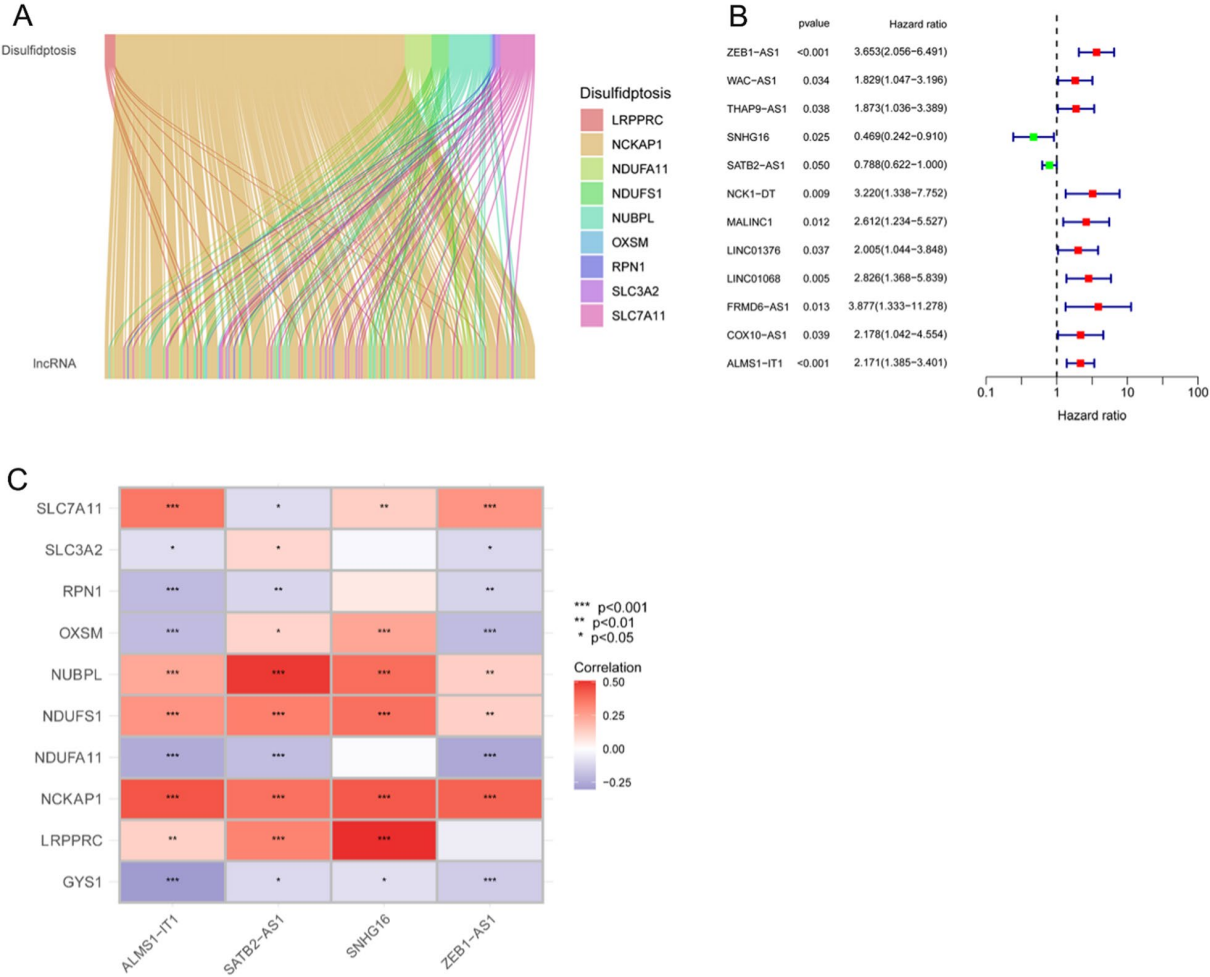
Identification of a disulfidptosis-associated lncRNA prognostic signature. **A** Sankey diagram of lncRNAs associated with disulfidptosis genes. **B** Forest plot of disulfidptosis-associated prognostic lncRNAs. **C** Correlation heat map between the lncRNAs involved in the model construction and disulfidptosis genes (*p < 0.05, **p < 0.01, ***p < 0.001)

The patients were randomly divided into training and test groups (Table 2). Following the Spearman correlation analysis of the co-expression of disulfidptosis genes with lncRNAs, 270 lncRNAs were identified (Fig. 1A). In addition, as shown in Fig. 1B, 12 prognostic lncRNAs were identified using UniCox analysis in the training group. As shown in Additional file 1: Fig. S1A, B, the four lncRNAs used to construct the model were identified using LASSO logistic regression analysis with the following equation (Coef retains three decimal places): risk score = (1.139) × Exp _ZEB1-AS1_ + (− 0.544) × Exp _SNHG16_ + (− 0.234) × Exp _SATB2-AS1_ + (0.478) × Exp _ALMS1-IT1_. In addition, we generated the heat maps of these four hub lncRNAs and disulfidptosis-associated genes (Fig. 1C).

**Table 2:** Clinicopathology characteristics of patients with COAD

Covariates		Whole group	Test group	Train group	*p* value
Age	≤ 65	158 (41.47%)	80 (42.11%)	78 (40.84%)	0.883
	> 65	223 (58.53%)	110 (57.89%)	113 (59.16%)	
Gender	Female	180 (47.24%)	83 (43.68%)	97 (50.79%)	0.1986
	Male	201 (52.76%)	107 (56.32%)	94 (49.21%)	
Stage	I	65 (17.06%)	31 (16.32%)	34 (17.8%)	0.373
	II	149 (39.11%)	71 (37.37%)	78 (40.84%)	
	III	102 (26.77%)	51 (26.84%)	51 (26.7%)	
	IV	54 (14.17%)	33 (17.37%)	21 (10.99%)	
	Unknow	11 (2.89%)	4 (2.11%)	7 (3.66%)	
T	T1	10 (2.62%)	5 (2.63%)	5 (2.62%)	0.752
	T2	67 (17.59%)	31 (16.32%)	36 (18.85%)	
	T3	260 (68.24%)	129 (67.89%)	131 (68.59%)	
	T4	44 (11.55%)	25 (13.16%)	19 (9.95%)	
M	M0	282 (74.02%)	139 (73.16%)	143 (74.87%)	0.1489
	M1	54 (14.17%)	33 (17.37%)	21 (10.99%)	
	Unknow	45 (11.81%)	18 (9.47%)	27 (14.14%)	
N	N0	228 (59.84%)	111 (58.42%)	117 (61.26%)	0.7004
	N1	87 (22.83%)	43 (22.63%)	44 (23.04%)	
	N2	66 (17.32%)	36 (18.95%)	30 (15.71%)

### Model evaluation and validation

**Fig. 2:**
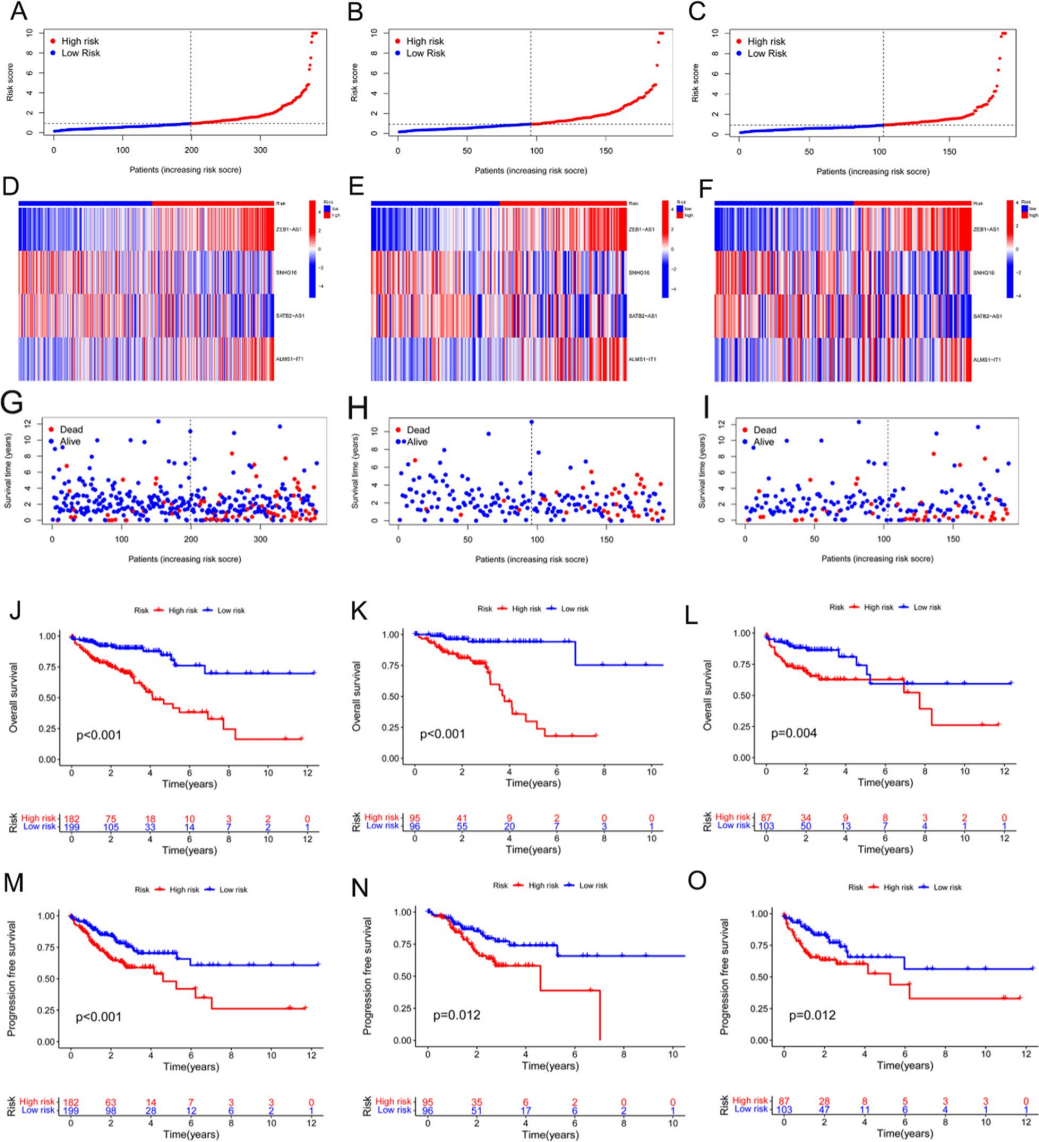
Validation of the prognostic function of the model. **A** The entire cohort of patients with COAD in the TCGA was divided into low- and high-risk sets based on the patients’ median risk scores. **B** The training group cohort of patients with COAD in the TCGA was classified into low- and high-risk sets based on the patients’ median risk scores. **C** The test group cohort of patients with COAD in the TCGA was grouped into low- and high-risk sets based on the patients’ median risk scores. **D–F** Heat maps of the expression results of the four lncRNAs between the samples from the two different risk groups in the whole, training, and test groups, respectively. **G–I** Life and death rate distributions of patients with COAD between the two risk groups in the whole, training, and test groups, respectively. **J–L** Overall survival curves corresponding to patients in the whole, training, and test groups, respectively. **M–O** Progression-free survival curves corresponding to patients in the whole, training, and test groups, respectively

Patients were divided into low- and high-risk groups based on their median risk scores. Figure 2A–C show the median risk scores, which were used to divide the patients in their respective groups into two risk groups: panels a, b, and, c represent the whole, training, and test groups, respectively. Figure 2D–F show the expression results of the four lncRNAs between the samples from the two different risk groups: d, e, and f represent the whole group, training set, and test set, respectively. Figure 2G–I show the patient survival status data between the two risk groups in the whole, training, and test groups, respectively. The survival results of the patients in the whole, training, and test sets showed that the model had a significant survival differentiation function, with patients in the high-risk group showing significantly poor OS (Fig. 2J–L). In addition, as shown in Fig. 2M–O, samples from the low-risk group tended to have higher favorable progression-free survival (PFS) than those from the high-risk group.

**Fig. 3:**
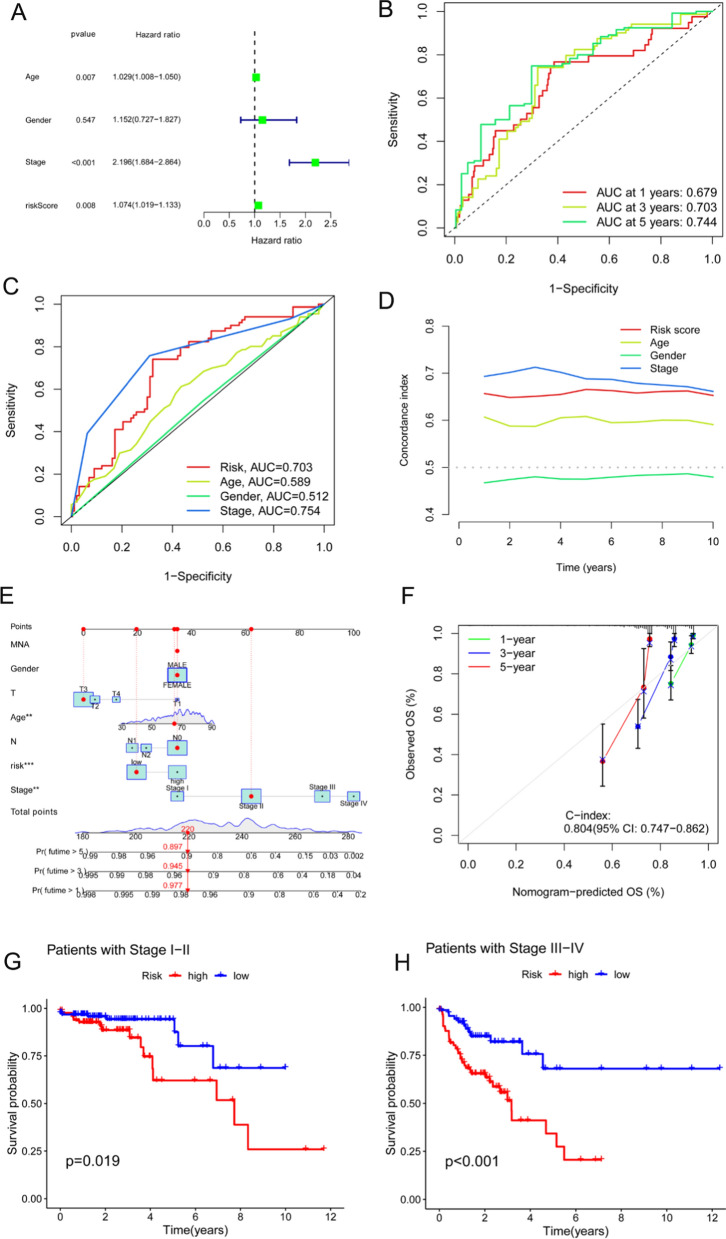
Comparison of models and other clinical indicators. **A** Forest plot for the univariate Cox regression analysis. **B** ROC curves of the model for the 1-, 3- and 5-year survival rates. **C** ROC curves of the model and other clinicopathological indicators. **D** C-index curves of the model and other clinicopathological variables. **E** Nomogram that combines the model and clinicopathological factors for the prediction of the 1-, 3-, and 5-year overall survival rates of patients with COAD. **F** Calibration curve to assess the concordance between the predicted OS rates and the actual OS rates. **G**, **H** Survival curves of patients with COAD in the early and advanced stages

Univariate regression analysis suggested that age, stage, and signature were independent prognostic indicators in patients with COAD (Fig. 3A). As shown in Fig. 3B, the area under curves (AUCs) of the 1-, 3-, and 5-year survival rates were 0.679, 0.703, and 0.744, respectively, indicating that the risk model had good predictive performance. In addition, as depicted in Fig. 3C, the model had an AUC of 0.703, which was superior to the clinicopathological indicators of age and sex in predicting the prognosis of patients with COAD. The concordance index in the model also outperformed the clinical indicators of age and sex (Fig. 3D).

As shown in Fig. 3E, a nomogram that included clinicopathological variables and signatures was developed to further identify the prognosis of patients with COAD. The results of the nomogram satisfactorily predicted the 1-, 3-, and 5-year prognostic probabilities of patients with COAD. The calibration curves shown in Fig. 3F indicate favorable concordance between the effective OS rates and the estimated 1-, 3-, and 5-year survival rates. Furthermore, according to the OS results of the model for patients with different clinical stages, the model was applicable to both early- and intermediate-to-late-stage COAD (Fig. 3G, H).

**Fig. 4:**
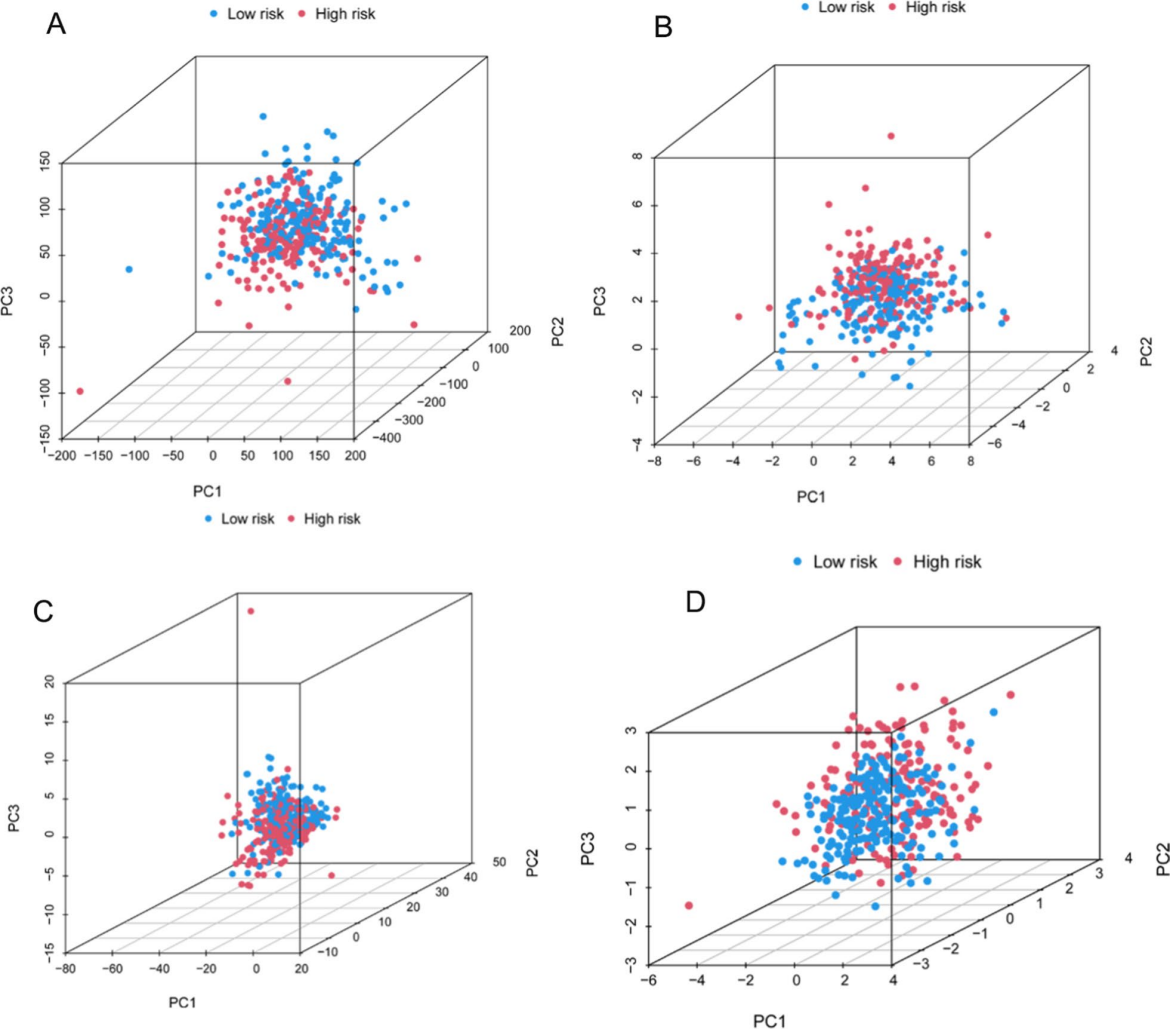
PCA. **A** PCA of all genes. **B** PCA of disulfidptosis-associated genes. **C** disulfidptosis-associated lncRNAs. **D** PCA of the model

Figure 4A–D show the results of sample division into two risk sets based on all genes, disulfidptosis-associated genes, disulfidptosis-associated lncRNAs, and the model, respectively. The results indicated that our constructed model displayed optimal discriminatory ability and was able to clearly distinguish between patients in the two risk sets.

### Enrichment analysis of DEGs

**Fig. 5:**
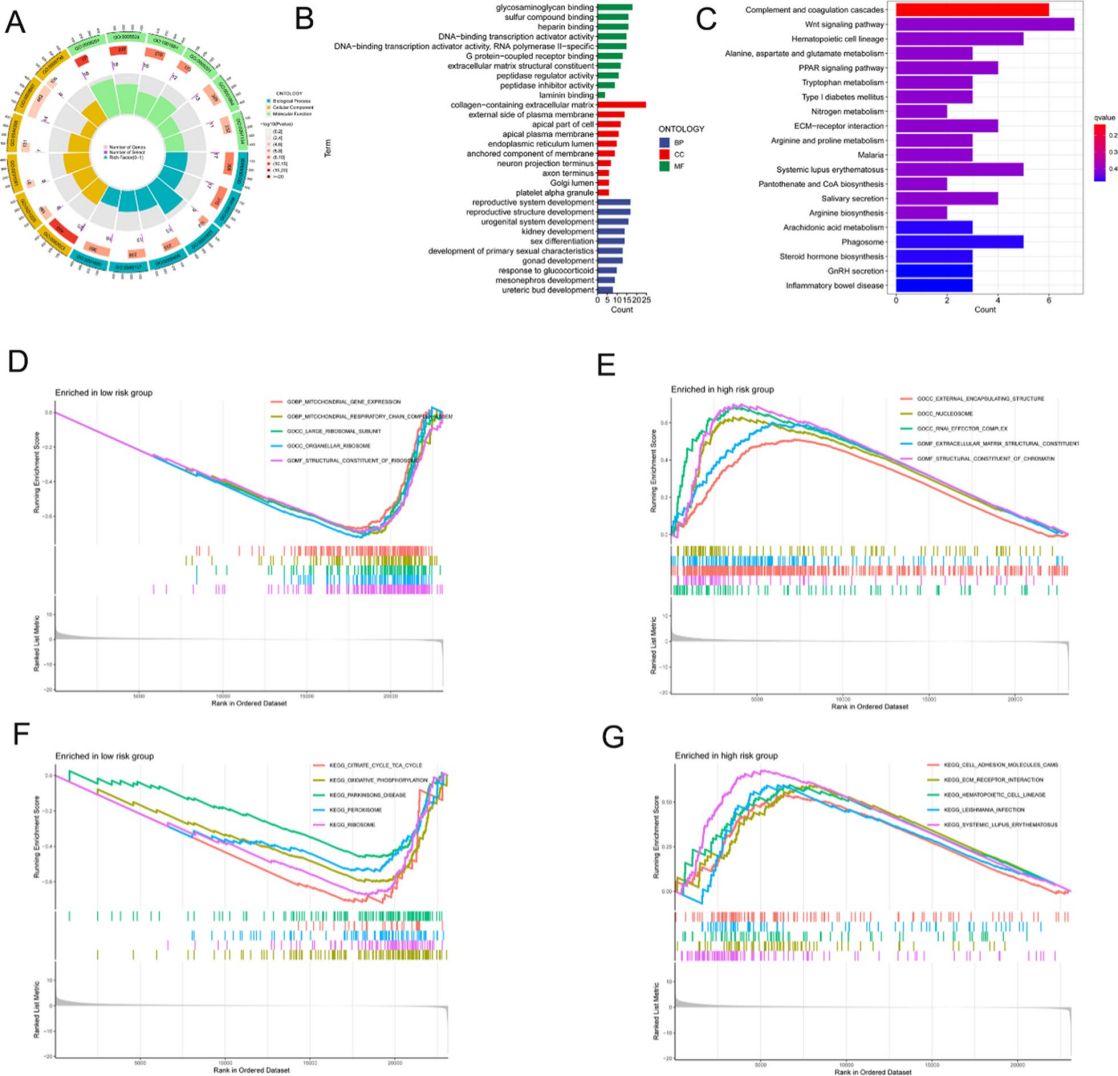
Enrichment analysis. **A**, **B** GO functional enrichment analysis. **C** KEGG pathway enrichment analysis. **D**, **E** GSEA functional enrichment analysis. **F**, **G** GSEA pathway enrichment analysis

The results of the GO functional enrichment analysis showed that disulfidptosis-associated DEGs were enriched in glycosaminoglycan binding, sulfur compound binding, external side of plasma membrane, collagen-containing extracellular matrix, and extracellular matrix structural constituents, suggesting that DEGs significantly contributed to disulfidptosis and cell structure disruption under conditions of glucose starvation (Fig. 5A, B). As shown in Fig. 5C, the results of the pathway analysis revealed that DEGs were mostly concentrated in the Wnt signaling pathway, which is an important mechanism for cancer function. In addition, enrichment of DEGs in several immune-related and amino acid metabolism-related pathways was identified.

The functional results of the GSEA are shown in Fig. 5D and E. In the low-risk set, the DEGs were mainly related to mitochondrial gene expression, mitochondrial respiratory chain assembly, and organellar ribosomes. In contrast, in the high-risk set, these were mainly enriched in the external encapsulating structure, extracellular matrix structural constituents, and structural constituents of chromatin. In the low-risk group, DEGs were abundant in the tricarboxylic acid cycle, whereas in the high-risk group, DEGs were associated with oxidative phosphorylation and were enriched in cell adhesion molecules and extracellular matrix receptor interactions (Fig. 5F, G).

### Immunocyte infiltration and somatic cell mutations

**Fig. 6:**
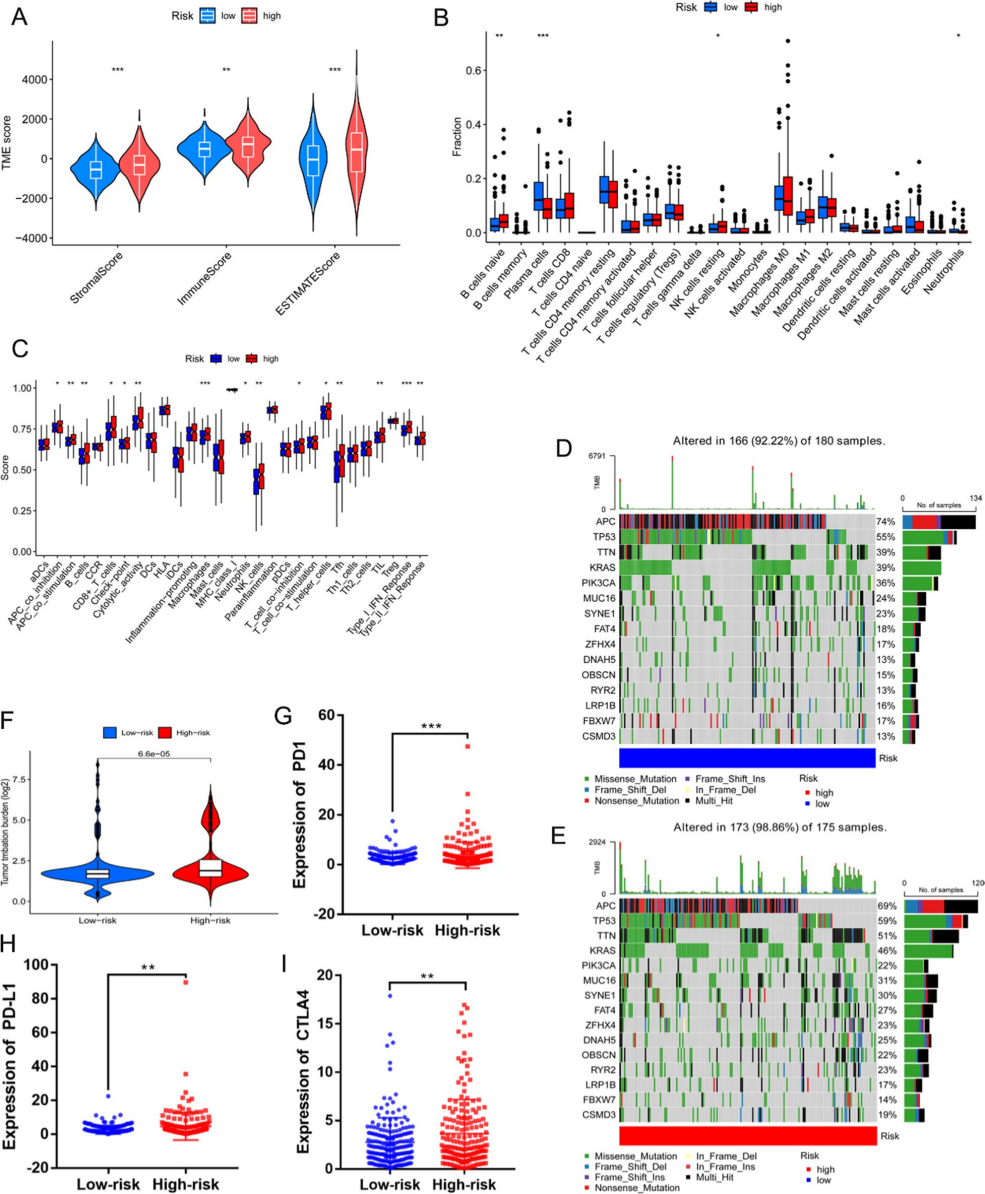
Analysis of patients’ tumor microenvironments and mutations. **A** Violin plot comparing the stromal, immune, and ESTIMATE scores between the high- and low-risk sets separately (ESTIMATE scores = stromal scores + immune scores). **B** Different immunocyte infiltration percentages. **C** ssGSEA scores for 16 immunocytes and 13 immune functions. **D**, **E** Waterfall plot of somatic mutations in the low- and high-risk groups. **F** Comparison of the TMB differences between the two risk groups. **G–I** Relative expression of PD1, PD-L1, and CTLA4 in the two risk groups (*p < 0.05, **p < 0.01, ***p < 0.001)

As shown in Fig. 6A, in the TIME analysis, the samples in the high-risk group tended to show higher stromal, immune, and ESTIMATE scores than those in the low-risk group, suggesting that stromal cells and immunocytes were more infiltrated and that tumor growth, invasion, and metastasis were more likely to occur in high-risk patients. The consequences of the immunocyte composition percentages in the two different risk sets are displayed in Fig. 6B, which shows that the fractions of naïve B cells and resting NK cells were higher in the high-risk group, whereas the proportions of plasma cells and neutrophils were higher in the low-risk group.

Figure 6C shows the correlation between the model, immunocytes, and their functions. The ssGSEA results revealed that the numbers of B cells, macrophages, neutrophils, CD8 + T cells, tumor-infiltrating lymphocytes (TIL), T follicular helper (Tfh) cells, and NK cells were significantly higher in the high-risk group than those in the low-risk group. Moreover, in terms of immune function, there was a significant correlation between antigen-presenting cell (APC) co-inhibition, T cell co-inhibition, cytolytic activity, checkpoints, APC co-stimulation, type I IFN responses, and type II IFN responses, which were significantly enriched in the high-risk group. These outcomes imply that immune functions were more active in the high-risk patient group than those in the low-risk patients group.

**Fig. 7:**
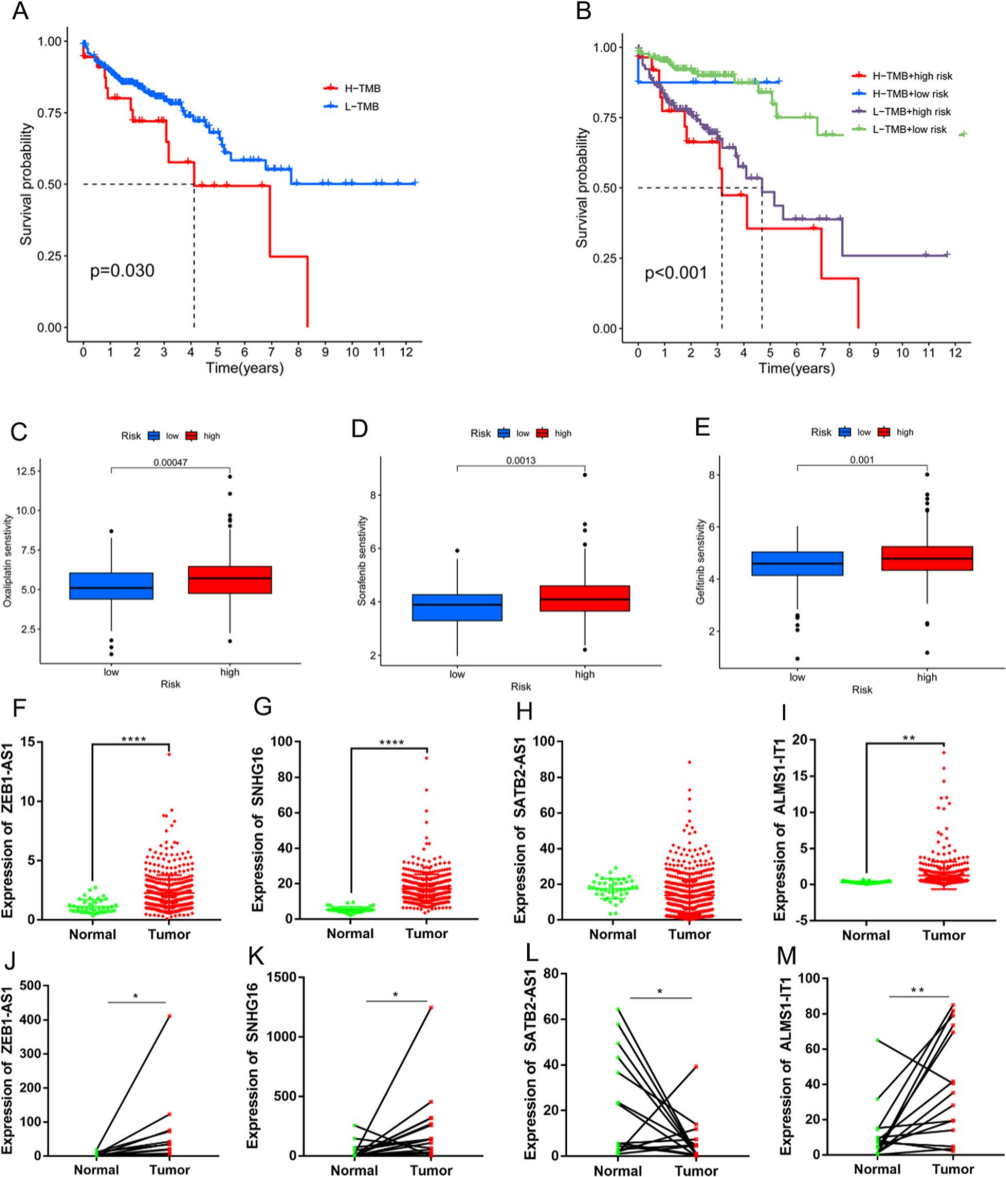
Validation of the model. **A** Survival analysis of two groups with high and low TMBs. **B** Survival analysis of two risk groups and two TMB groups. **C–E** Sensitivity comparisons of commonly used front-line chemotherapeutic agents between two groups. **F–I** Relative expression levels of ZEB1-AS1, SNHG16, SATB2-AS1, and ALMS1-IT1 in cancer and paracancerous samples from patients with COAD in the TCGA, respectively. Relative expression levels of ZEB1-AS1, SNHG16, SATB2-AS1, and ALMS1-IT1 in cancer and paracancerous samples from patients with COAD in the TCGA, respectively. **J–M** Relative expression levels of ZEB1-AS1, SNHG16, SATB2-AS1, and ALMS1-IT1 in COAD and paired non-cancerous tissue samples, respectively, from the Affiliated Hospital of Qingdao University (*p < 0.05, **p < 0.01, ***p < 0.001, ****p < 0.0001)

The distributions of the somatic mutations in patients with COAD in the two risk sets are shown in Fig. 6D, E. The frequency of mutations was higher in patients with COAD in the high-risk group than that in patients with COAD in the low-risk group. In addition, the TMB results observed in the high-risk group were greater than those observed in the low-risk group (Fig. 6F), and three immune checkpoints were also expressed at higher levels in the high-risk group (Fig. 6G–I) than those in the low-risk group, suggesting that the samples in the high-risk group were responsive to immune checkpoint inhibitors (ICIs). Furthermore, patients with lower TMBs frequently had better survival rates (Fig. 7A), whereas those with higher TMBs and those in the high-risk group had the worst prognoses (Fig. 7B).

### Treatment effectiveness prediction

When comparing the drug sensitivity data from clinical trials and common clinical applications, considerable differences in the IC50 values of multiple chemotherapeutic agents between the two risk groups were observed (Fig. 7C–E show the first-line clinical use data for patients with COAD, and the remaining drug results are shown in Additional file 1: Fig. S2–4).

### Expression of the lncRNAs in the model

Figure 7F–I show the results of four lncRNAs identified in cancerous and non-cancerous tissues from patients with COAD in the TCGA database; the expression levels of ZEB1-AS1, SNHG16, and ALMS1-IT1 were higher in tumor samples than those in normal samples (*p* < 0.05), whereas the reverse was observed for SATB2-AS1. Figure 7J–M show the results of the expression validation of four lncRNAs in a cohort of 15 patients (normal tissues:15, paracancerous tissues: 15) with COAD at our hospital, which showed that ZEB1-AS1, SNHG16, and ALMS1-IT1 were expressed at higher levels in tumor samples than those in the corresponding paracancerous samples (*p* < 0.05), whereas SATB2-AS1 was upregulated in the paracancerous samples (*p* < 0.05).

## Discussion

As one of the most frequent neoplasms, the incidence of COAD gradually increases with changes in diet and lifestyle habits [21, 22]. Despite significant progress in the understanding of the biological mechanisms underlying COAD, and although the 5-year survival rate of patients with COAD has improved significantly, the prognosis of advanced COAD remains extremely poor [23, 24]. Therefore, improving COAD screening and early diagnosis methods, improving treatment modalities, and reducing recurrence and metastasis in patients will require further continuous analysis and exploration of the mechanisms underlying the development of COAD.

A recent study identified a new type of cell death independent of cuproptosis and ferroptosis denoted as “disulfidptosis,” which is a rapid mode of cell death caused by disulfide stress induced by excessive intracellular cystine accumulation [5]. In glucose-deficient SLC7A11-high cancer cells, massive accumulation of disulfide molecules leads to abnormal disulfide bonding between actin cytoskeletal proteins, disrupting their organization and ultimately leading to actin network collapse and cell death [5]. Nine other disulfidptosis-related genes have been identified through sequencing: *GYS1, NDUFS1, OXSM, LRPPRC, NDUFA11, NUBPL, NCKAP1, RPN1*, and *SLC3A2* [5]. Therefore, disulfidptosis is a potential target for cancer therapy. Recently, studies have been conducted to predict the survival of patients with cancer by generating lncRNA prediction signatures associated with cuproptosis and ferroptosis [25–28]. However, disulfidptosis-related lncRNAs have not yet been identified as prognostic markers. This is the first study to develop a prognostic risk model based on four disulfidptosis-related lncRNAs to systematically study the tumor and immune microenvironment to indicate immunotherapy and chemotherapy, as well as to guide patient prognosis.

In the current study, using information from the TCGA database, co-expressed lncRNAs were first identified based on 10 disulfidptosis genes. Then, prognosis-related lncRNAs were acquired via UniCox analysis, and more accurate prognostic lncRNAs were obtained with a LASSO regression for model construction. The reliability of the model was confirmed by validating the risk sets of the two clinicopathological indicators. It was found that samples with lower risk sets had better OS and PFS. The enrichment analysis of DEGs associated with disulfidptosis revealed associations with aspects of sulfur compound binding and disruption of cell structure, immune-related and amino acid metabolism-related pathways, and Wnt signaling. In their study, Liu et al. showed that adding more cystine to a glucose-free medium caused NADPH overconsumption and induced actin cytoskeleton protein disulfide bond cross-linking and cytoskeleton contraction in SLC7A11 low-expressing cells, ultimately inducing disulfidptosis [5], which is consistent with our findings.

The TIME is composed of tumor and stromal cells and the extracellular matrix, and it is thought to be a significant factor in tumor cell invasion, migration, adhesion, colonization, and neovascularization [29]. Various innate immunocytes (macrophages, dendritic cells, NK cells, and others) and adaptive immunocytes (T cells, B cells, and others) are present in the TIME and influence tumor development [30]. In this study, it was found that patients in the high-risk group had more stromal cells and immunocytes and worse tumor progression than those in the low-risk group. It has been demonstrated that tumor-infiltrating macrophages produce several mediators in the TIME to facilitate tumor proliferation, metastasis, and invasion, leading to poor prognosis [31]. In addition, one study reported that increased infiltration of macrophages, neutrophils, and Tfh cells in adenomatous colon polyps was correlated with the degree of malignancy, which provides guidance for colon cancer treatment [32]. Moreover, Ahmadzadeh et al. showed that the tumor microenvironment, including CD8 + cells and TIL, increases the growth of melanoma. Consistently, our study demonstrated that patients in the high-risk group had more neutrophils, CD8 + T cells, Tfh, and TIL, in addition to worse prognoses than those in the low-risk group. In addition, a recent study found a significant association between tumor-infiltrating plasma cells increased and good prognosis in patients with ovarian cancer, which is consistent with our results that patients in the low-risk group tend to have a better prognosis [33].

The study of B cells is controversial, with previous studies demonstrating their antitumor role in tumor immunity; however, these have also been shown to promote tumorigenic effects [34]. In this study, we demonstrated the tumor-promoting role of B cells in a high-risk group of patients. Notably, cancer patients’ responses to ICIs treatments are related to the quantity and quality of T, NK, and B cells in the TIME [35].

ICIs have shown exciting results in non-small cell lung cancer, melanoma, colon cancer, and others [36–38]. According to some clinical trials, ICIs can still be useful for patients with malignancies who have already received chemotherapy or targeted therapy [39]. ICIs, including PD-1, PD-L1, and CTLA-4 inhibitors, provide treatment options for patients with malignant tumor [40]. The TMB is determined according to the total number of mutations per megabase in a tumor tissue sample, and studies have demonstrated that the higher the TMB, the more significant the overall survival benefit for patients with high TMB and those treated with ICIs [41, 42]. Corroborating this, the current study revealed that patients in the high-risk group had higher TMBs and expression of immune checkpoints, indicating that these patients may have a higher response rate to ICIs treatment than those in the low-risk group.

An increasing body of research suggests that lncRNAs play a predominantly oncogenic role in cancer [43, 44]. Due to their high tissue specificity and ease of detection in human bodily fluids, lncRNAs have great potential as diagnostic biomarkers and therapeutic targets [45]. Four disulfidptosis-related lncRNAs were identified in this study, including ZEB1-AS1, SNHG16, ALMS1-IT1, and SATB2-AS1. Several studies have demonstrated that ZEB1-AS1 plays an important role in regulating the proliferation, apoptosis, migration, invasion, and drug resistance of colon cancer cells [46, 47]. Christensen et al. demonstrated that SNHG16, which is upregulated in colon cancer, is regulated by Wnt signaling and contributes to the progression of COAD [48]. In addition, ALMS1-IT1 has been shown to be associated with immune infiltration in COAD and ferroptosis; thus, this lncRNA could be used as a biomarker for the prognosis of COAD [49, 50]. Furthermore, SATB2-AS1 inhibits COAD metastasis by activating SATB2 through DNA demethylation in the SATB2 promoter region. Additionally, SATB2-AS1 could inhibit T helper type 1 cells and immunocyte density in COAD [51].

In summary, lncRNA models associated with disulfidptosis independently predicted the prognosis of patients with COAD and were significantly associated the TIME of patients as well as their sensitivity to immunotherapy and chemotherapy. We aimed to provide a rationale for the underlying mechanisms of disulfidptosis-related lncRNAs in COAD and their effects on clinical therapies. However, this investigation has some limitations. In addition to the relatively finite sample size, the model requires further biological validation.

### Supplementary Information

**Additional file 1: Figure S1.** Cross-validation curves of the LASSO regression. **Figure S2–4.** Comparison of the sensitivity of the experimental and other clinical chemotherapeutic agents between the two groups.

## Data Availability

Publicly available datasets were analyzed for this study, and further information can be obtained from the corresponding author upon reasonable request.

## Abbreviations

COAD: Colon adenocarcinoma
TIME: Tumor immune microenvironment
UniCox: Univariate Cox
OS: Overall survival
LASSO: Least absolute shrinkage and selection operator
ROC: Operating characteristic
C-index: Consistency index
PCA: Principal component analysis
DEGs: Differentially expressed genes
GO: Gene ontology
KEGG: Kyoto Encyclopedia of Genes and Genomes
GSEA: Gene set enrichment analysis
SsGSEA: Single-sample GSEA
TMB: Tumor mutation burden
IC50: Inhibitory concentration
qRT-PCR: Quantitative real-time PCR
cDNA: Complementary DNA
PFS: Progression-free survival
AUCs: Area under curves
TIL: Tumor-infiltrating lymphocytes
Tfh: T follicular helper
APC: Antigen-presenting cell
ICIs: Immune checkpoint inhibitors
