# Supplementary material for: Disulfidptosis-associated long non-coding RNA signature predicts the prognosis, tumor microenvironment, and immunotherapy and chemotherapy options in colon adenocarcinoma
Source: Cancer Cell Int. 2023 Sep 27;23:218. doi: 10.1186/s12935-023-03065-8 (PMC10523716; doi:10.1186/s12935-023-03065-8)
Supplement: Supplementary file 1 — Additional file 1: Figure S1. Cross-validation curves of the LASSO regression. Figure S2–4. Comparison of the sensitivity of the experimental and other clinical chemotherapeutic agents between the two groups. [file 12935_2023_3065_MOESM1_ESM.docx]

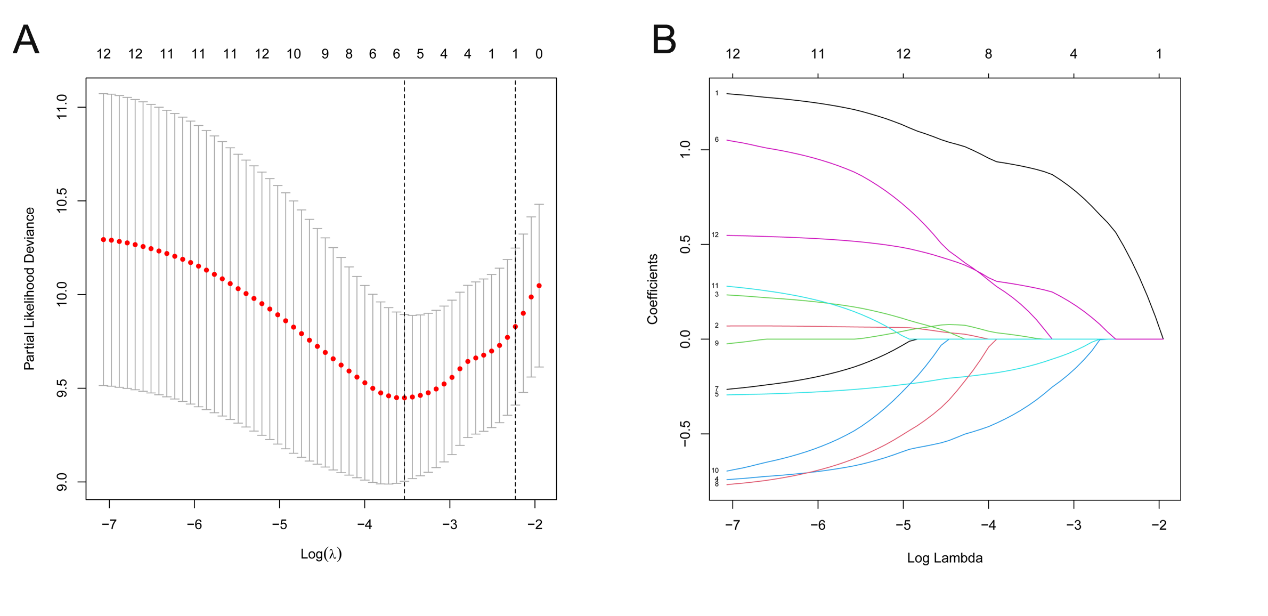


**Figure S1. Cross-validation curves of the LASSO regression.**


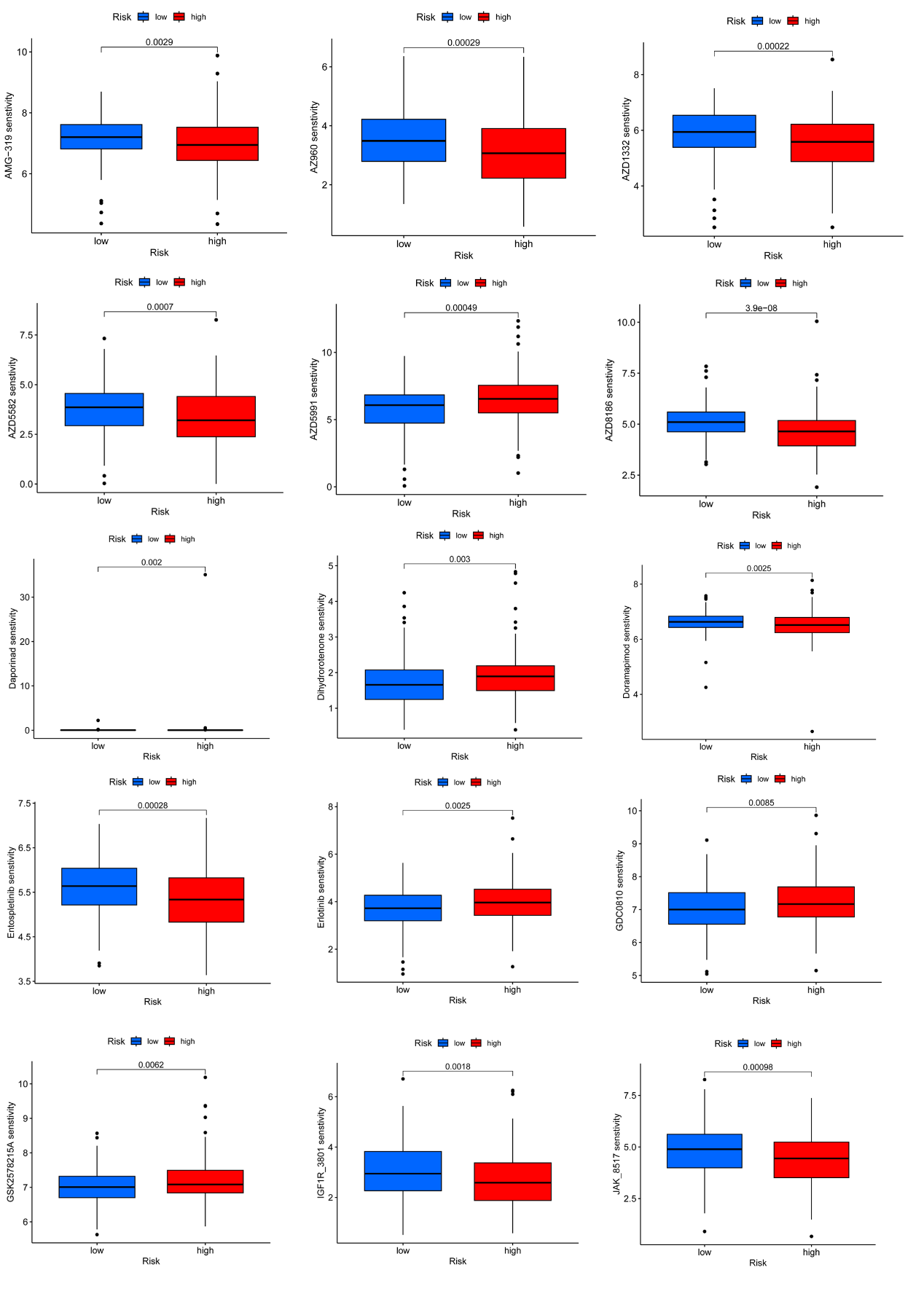


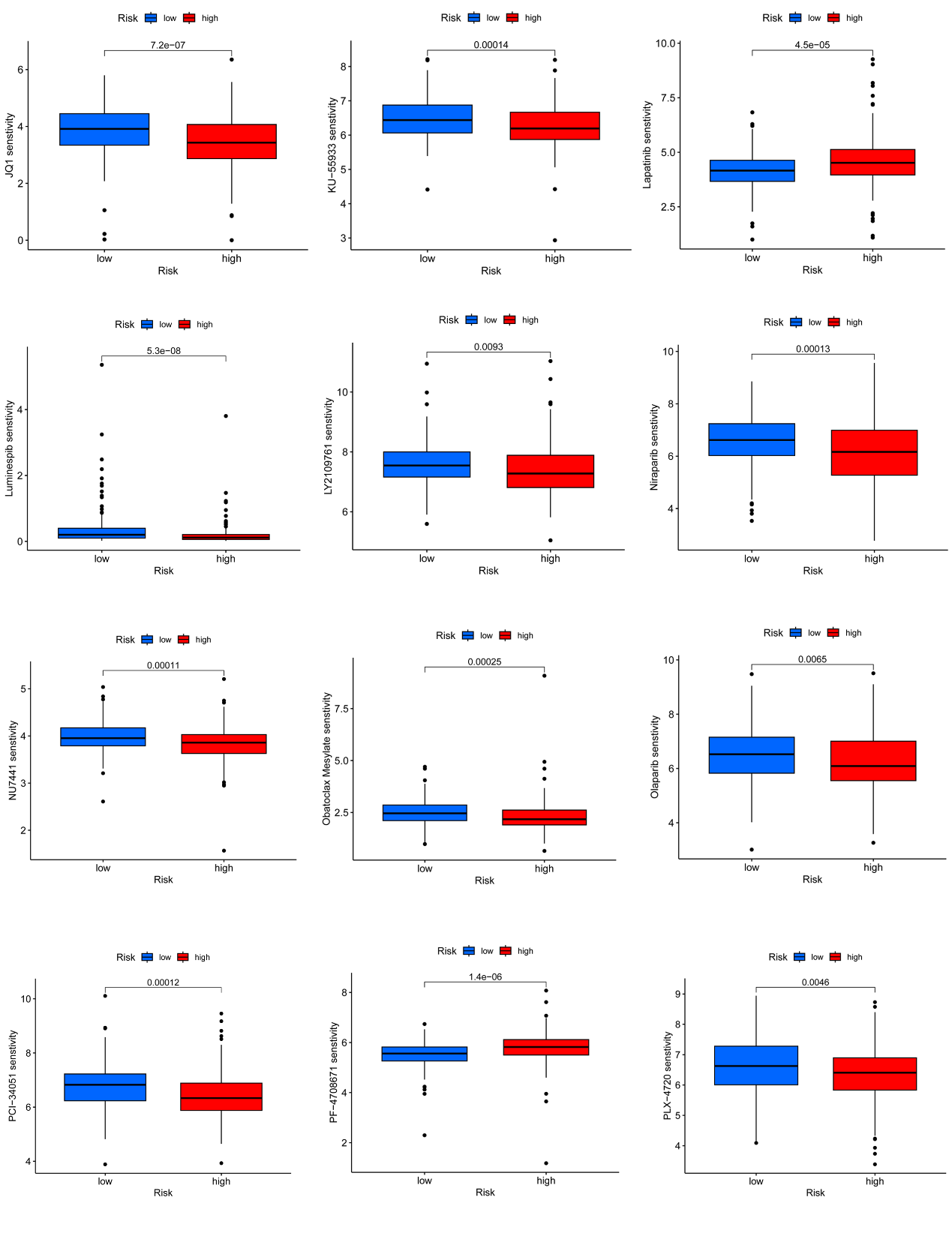


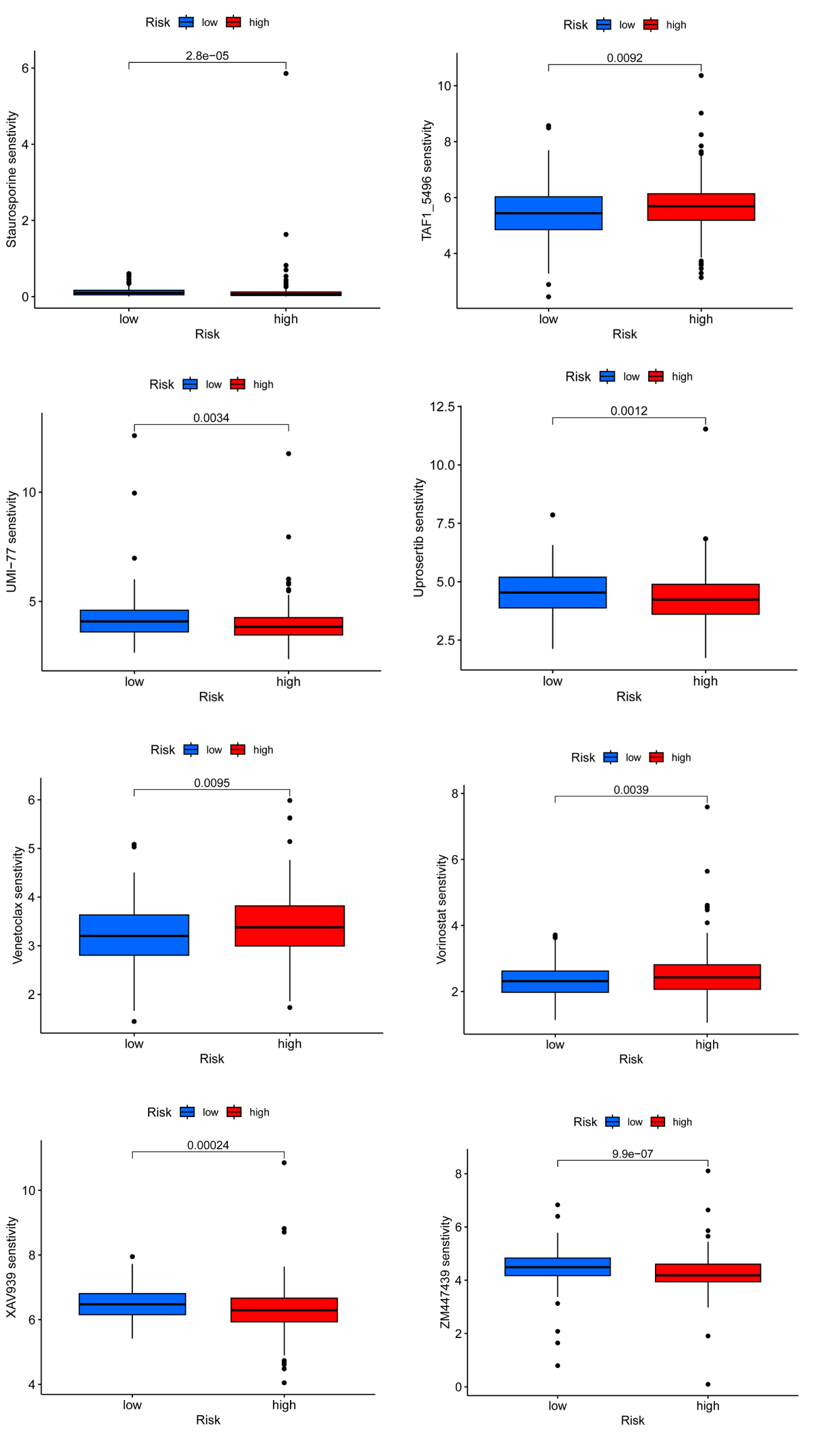


**Figure S2–4. Comparison of the sensitivity of the experimental and other clinical chemotherapeutic agents between the two groups**
